# Studying synaptic efficiency by post-hoc immunolabelling

**DOI:** 10.1186/1471-2202-14-127

**Published:** 2013-10-18

**Authors:** Jorge Ramírez-Franco, Beatris Alonso, David Bartolomé-Martín, José Sánchez-Prieto, Magdalena Torres

**Affiliations:** 1 Departamento de Bioquímica, Facultad de Veterinaria, Universidad Complutense, Madrid 28040, Spain

**Keywords:** *Post-hoc* immunocytochemistry, FM1-43, Synaptic vesicle exocytosis, RIM1α, Munc13-1

## Abstract

**Background:**

In terms of vesicular recycling, synaptic efficiency is a key determinant of the fidelity of synaptic transmission. The ability of a presynaptic terminal to reuse its vesicular content is thought to be a signature of synaptic maturity and this process depends on the activity of several proteins that govern exo/endocytosis. Upon stimulation, individual terminals in networks of cultured cerebellar granule neurons exhibit heterogeneous exocytic responses, which reflect the distinct states of maturity and plasticity intrinsic to individual synaptic terminals. This dynamic scenario serves as the substrate for processes such as scaling, plasticity and synaptic weight redistribution. Presynaptic strength has been associated with the activity of several types of proteins, including the scaffolding proteins that form the active zone cytomatrix and the proteins involved in presynaptic exocytosis.

**Methods:**

We have combined fluorescence imaging techniques using the styryl dye FM1-43 in primary cultures of cerebellar granule cells with subsequent *post-hoc* immunocytochemistry in order to study synaptic efficiency in terms of vesicular release. We describe a protocol to easily quantify these results with minimal user intervention.

**Results:**

In this study we describe a technique that specifically correlates presynaptic activity with the levels of presynaptic markers. This method involves the use of the styryl dye FM1-43 to estimate the release capacity of a synaptic terminal, and the subsequent *post-hoc* immunolabelling of thousands of individual nerve terminals. We observed a strong correlation between the release capacity of the nerve terminal and the levels of the RIM1α but not the Munc13-1 protein in the active zone.

**Conclusions:**

Our findings support those of previous studies and point out to RIM1α as a crucial factor in determining synaptic efficiency. These results also demonstrate that this technique is a useful tool to analyse the molecular differences underlying the heterogeneous responses exhibited by neuronal networks.

## Background

Presynaptic active zones (AZ) are specialized axonal sites of fusion that mediate neurotransmitter release into chemical synapses. A complex network of proteins is assembled at axonal sites that generate the so-called cytomatrix at the active zone (CAZ). These proteins interact with other proteins located either at the presynaptic plasma membrane or at vesicular membranes that regulate Ca^2+^-dependent fusion of synaptic vesicles. The different stages of the synaptic vesicular cycle (docking, priming, exocytosis and compensatory endocytosis) are orchestrated by distinct subsets of proteins, and the amount and interaction of these different proteins are thought to be crucial in determining presynaptic strength. The capacity of a given synapse to efficiently reuse synaptic vesicles has been proposed as a hallmark of maturation, and differences in vesicular reuse appear to underlie the enormous variability of responses observed in cultured neuronal networks [1]. Remodelling of the active zone through changes in protein content or post-translational modifications has been linked with several crucial mechanisms involved in synaptic physiology, including presynaptic potentiation/depression, homeostatic synaptic scaling, synaptic silencing and synaptic weight redistribution [2-7].

In the present study, we focused on RIM1α as this protein is a key organizer of the active zone and it interacts directly or indirectly with all other known active zone proteins, including Rab3A and Munc13 [8]. Indeed, the RIM proteins are required for synaptic vesicle priming and both short- and long-term synaptic plasticity [9-12]. These RIM’s tether Ca^2+^ channels to the presynaptic active zone [13] and activate vesicle priming by reversing the autoinhibitory homodimerization of Munc13 [14]. Moreover, the RIM1/2 content is linearly associated with release probability and the size of the active zone [7]. Consistent with this central role, Rim deletion prevents neurotransmitter release [13]. In addition to RIM1α, we also analyzed Munc13-1, given the key role of Munc13 proteins in priming synaptic vesicles to a fusion-competent state [15] and in short-term potentiation of transmitter release [15-17].

*Post-hoc* immunocytochemistry and immunohistochemistry have previously been used to study synaptic and neuronal function [1,18-20], yet to date, no detailed method to perform and analyse these experiments has been described. Here, we present a method that combines the assessment of presynaptic function in primary cultures of cerebellar granule neurons by monitoring synaptic vesicle recycling using the styryl dye FM1-43 with subsequent *post-hoc* immunocytochemistry. In addition, we describe a semi-automated protocol to easily quantify the data obtained, which enables the levels of immunoreactivity (IR) to be correlated with synaptic efficiency.

## Methods

### FM1-43 live cell imaging

**Figure 1:**
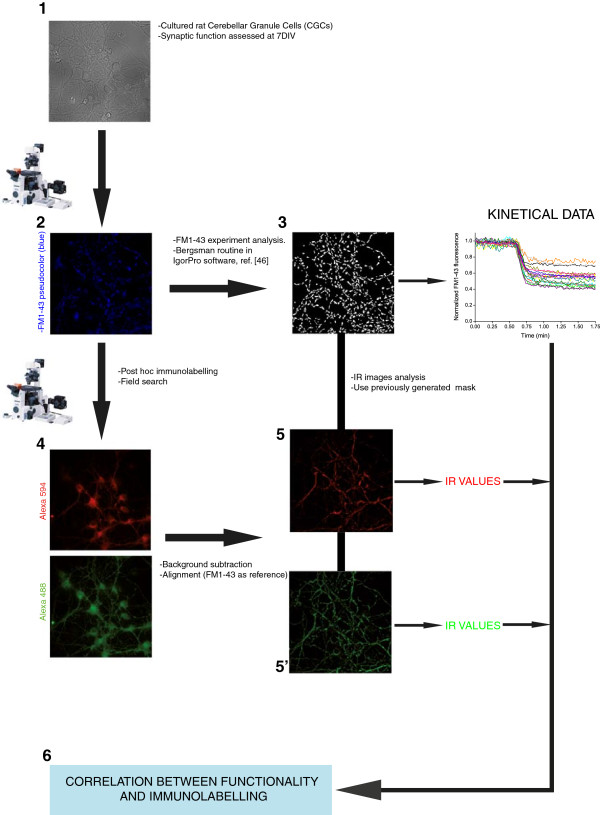
**Graphical abstract of FM1-43 experiments and subsequent post-hoc immunolabelling.** Each number represents a different step in the protocol detailed throughout the paper. Briefly, 7DIV cultured cerebellar granule cells **(1)** were used to assess synaptic efficiency by means of FM1-43 experiments **(2)**. Then, experiment analysis **(3)** was performed using the routine described in [46]. Cells were then labelled with antibodies against different presynaptic proteins **(4)**. After alignment and analysis of the acquired images **(5)** the immunoreactivity measurements were correlated with the data obtained in the functional experiment **(6)**.

To assess presynaptic activity we used cultures of cerebellar granule neurons that are largely populated by glutamatergic neurons [21-23]. All experiments were carried out in accordance with the guidelines established by the National Council on Animal Care and were approved by the local Animal Care Committee of the Universidad Complutense de Madrid (UCM, Madrid, Spain) following European Communities Council Directive of 22 September 2010 (2010/63/EU). Every possible effort was made to minimize animal suffering and the number of animals used. Cultures were prepared as described previously [1] and the cells were seeded at a final density of 3 × 10^5^ cells per coverslip. Synaptic efficiency was assessed after 7 days *in vitro* (DIV: Figure 1, step 1) as follows. Briefly, cells were incubated for 10 minutes at 37°C in a calcium-free, low-potassium buffer (140 mM NaCl, 5 mM KCl, 5 mM NaHCO_3_, 1.2 mM NaH_2_PO_4_, 1 mM MgCl_2_, 10 mM glucose, 10 mM HEPES [pH 7.4]), and then for 90 seconds at 37°C with 10 μM FM1-43 (Invitrogen) in high-potassium buffer (95 mM NaCl, 50 mM KCl, 1 mM MgCl_2_, 5 mM NaHCO_3_, 1.2 mM NaH_2_PO_4_, 1.33 mM CaCl_2_, 10 mM glucose, 10 mM HEPES [pH 7.4]). The coverslips were then mounted in a PH5 perfusion chamber (Warner instruments) and the surface-bound dye was removed by perfusion of a calcium-free low-potassium buffer for 10 minutes. For subsequent immunocytochemical experiments we did not use any kind of vacuum grease as the superficial tension of the remaining aqueous interphase of the coverslip was sufficient to prevent solution leakage. Perfusion into the chamber was performed using a VC6 perfusion system (Warner instruments) and all solutions were maintained at 37°C using a TC-344B temperature controlling system (Warner instruments). Baseline measurements were acquired over 30 seconds while perfusing low-potassium medium, after which the cells were stimulated for 10 seconds with the high-potassium medium, resulting in dye unloading. Short stimulation periods were chosen to minimize the unreliable homogeneity of responses that can occur during overstimulation of neuronal networks. During the experiment images were acquired at a rate of 1 Hz using a Nikon Eclipse TE2000-S microscope equipped with a Nikon CFI Plan Apo VC 60× Oil objective 1.4 (NA) and a CCD camera (iXon^EM^ + DU885, Andor Technology). A 479 nm monochromator was used for excitation and the emitted light was collected using a fluorescein isothiocyanate (FITC) filter. Once the imaging period was over, the cells were maintained at rest by perfusing low-potassium buffer and several phase contrast images were acquired at different magnifications (60×, 40× and 20×) to serially reconstruct the exact field used in the experiment at a lower magnification (Additional file 1: Figure S1). This serial reconstruction allowed us to locate the corresponding field after performing immunocytochemical experiments. The protocol used is described below and corresponds to step 2 in Figure 1; **1 Accommodation period:** Maintain the cells for 10 minutes in low-potassium buffer (140 mM NaCl, 5 mM KCl, 5 mM NaHCO_3_, 1.2 mM NaH_2_PO_4_, 1 mM MgCl_2_, 10 mM glucose, 10 mM HEPES [pH 7.4]) at 37°C; **2 Loading step:** Incubate the cells with the loading solution in high-potassium buffer in order to stain the synaptic vesicles (95 mM NaCl, 50 mM KCl, 1 mM MgCl_2_, 5 mM NaHCO_3_, 1.2 mM NaH_2_PO_4_, 1.33 mM CaCl_2_, 10 mM glucose, 10 mM HEPES [pH 7.4] and 10 μM FM1-43 [Invitrogen]) at 37°C; **3 Mounting:** Mount the coverslip in the perfusion chamber. It is important to do this rapidly to prevent the coverslip from drying. For a scheme to clarify this step, see Cheung and Cousin, 2011 [24]. Several different commercial and custom-built chambers can be used; **4 Wash-out period:** Once mounted in the chamber, wash the excess of externally bound dye from the coverslip by perfusion of a low-potassium, calcium-free buffer (140 mM NaCl, 5 mM KCl, 5 mM NaHCO_3_, 1.2 mM NaH_2_PO4, 1 mM MgCl_2_, 10 mM glucose, 10 mM HEPES [pH 7.4]) at 37°C for 10 minutes at a constant flow of 1 ml/min; **5 Imaging of the SV cycle:** Image acquisition can be performed at different frequencies. In this protocol we recorded at a rate of 1 frame per second (1 Hz), with the excitation wavelength set at 479 nm, and we collected the emitted light through a FITC filter (520/540 nm). We acquired 30 frames to define the baseline period (a shorter baseline period can also be used but is less optimal to ensure baseline stability) and then stimulated for the desired period by perfusing with a high-potassium solution. Shorter stimulation periods are associated with greater heterogeneity in the whole population of responses. The images were acquired with a CCD camera operating at 14 bits (iXon^EM^ + DU885, Andor Technology). Once image acquisition has ended, several images of the exact field imaged in the experiment and of the surrounding regions are collected at lower magnification. This is useful to build a serial reconstruction that helps define the exact field visualized during the experiment; **6 Coverslip removal:** Remove the microscope adapter without disassembling the perfusion chamber and extract the upper coverslip from the chamber. Then, unscrew the chamber from the microscope adapter and press the lower coverslip gently onto one of its edges with fine-tip surgical tweezers while pulling the entire chamber away from the platform. In this way it is possible to retrieve the coverslip almost intact from the slot in the platform. The subsequent immunocytochemical experiment should be commenced immediately and the collected data stored in a multidimensional *.TIFF file.

### *Post-hoc* immunocytochemistry

#### Antibodies

The following antibodies were used in the present study: a mouse monoclonal anti-Munc13-1, (1:1000; ref. 126 111 Synaptic Systems); a rabbit polyclonal anti-RIM1 (1:400; ref. 140 003 Synaptic Systems); and a guinea pig polyclonal anti-CB1R (1:300; ref. CB1-GP-Af530, Frontier Institute Co., Ltd.), the latter used exclusively as a presynaptic marker to obtain a positive linear relationship between the channels. To check for the specificity of the antibodies we performed western blot experiments as previously described [1]. Both antibodies recognized a single band with the expected molecular weight for both proteins (Additional file 2: Figure S2). The western blot protocol is described in Additional file 3.

#### Labelling procedures

**Figure 2:**
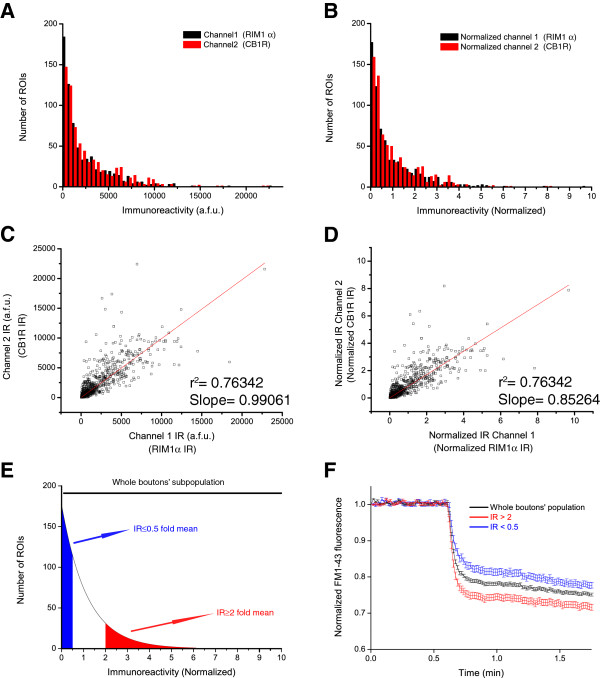
**Alignment of the different channels used for *post-hoc *ICC.** Measured IR values for two presynaptic markers in each one of the individual ROIs: channel 1 (Alexa 488, anti-RIM1α in this case) and channel 2 (Alexa 594, anti-CB1R in this case) are shown in a.f.u (arbitrary fluorescence units) **(A)** or normalized to their mean IR value **(B)**. Distribution of IR values of channel 1 (black bars) and channel 2 (red bars) in arbitrary fluorescence units **(C)** and normalized to mean levels **(D)**. **E)** Graphical representation of the distribution of normalized IR values (<0.5 and >2 fold mean in blue and red, respectively) through the whole population of synaptic boutons. **F)** Normalized FM1-43 unloading kinetics of the three groups of synaptic boutons according to their RIM1α IR levels, means ± S.E.M. are plotted. Data from a single experiment (n = 671 boutons for the whole population trace; n = 96 boutons for IR levels >2, red trace and n = 231 boutons with IR levels < 0.5 mean value).

To study the potential relationship between vesicular release efficiency and the levels of presynaptic proteins involved in neurotransmitter release, we selected two proteins with well-established roles in neurotransmitter release: Munc13-1 [16,25] and RIM1α [13,26]. Even when the amount of protein and the intensity of immunoreactivity are not linearly related [27], immunocytochemical analysis is the only possible means of exploring the differences in protein content in subcellular compartments and relating these to functional parameters (in this case vesicular release). Moreover, several studies have used IR intensity as a means of estimating relative amounts of synaptic proteins [2-4,28,29], although some changes in synaptic protein composition that can be demonstrated by proteomic techniques can also be detected by semi-quantitative analysis [30]. After removing the coverslips from the platform they were rinsed once in PBS at 37°C in order to detach the cell debris that might have formed during the experiment. The coverslips were then fixed in 4% paraformaldehyde for 15 minutes at room temperature (RT), washed twice for 5 minutes in PBS and then permeabilized for 6 minutes with PBS-0.2% Triton X-100 at RT. Before immunostaining, the cells were blocked for 1 hour at 37°C in a solution containing PBS-0.05 Triton X-100, 5% donkey serum and 5% goat serum (both from Jackson ImmunoResearch). The cells were then incubated overnight (o/n) at 4°C with the primary antibodies. Double immunocytochemistry for presynaptic markers is recommended even when only one marker will be used for further analysis, and staining in two different channels is useful to obtain a linear relationship between markers. Positive slope values (close to 1) for this relationship and strong correlation values are *bona fide* indicators of the validity of individual experiments (see section FM1-43 experiment and *post-hoc* immunocytochemistry and Figure 2). After exposure to the antibodies the cells were washed 5 times for 5 minutes each, and they were then incubated with Alexa (Molecular Probes, Invitrogen)-labeled secondary antibodies (alexa fluor 488 anti-mouse, goat 1:200; alexa fluor 488 anti-rabbit, goat 1:200 and alexa fluor 594 anti-guinea pig, goat 1:200) for 1 hour at 37°C. After washing, the coverslips were mounted with Prolong Antifade with DAPI (Invitrogen). The protocol used is described below:

**1 Fixation and blocking:** After the removal of the coverslip, rinse it thoroughly in PBS at 37°C and fix by immersion in 4% paraformaldehyde for 15 minutes. Wash twice in PBS and permeabilize the cells for 6 minutes in PBS-0.2% Triton X-100 if immunodetection of intracellular epitopes is required. Wash for 5 minutes in PBS and using a P100 pipette, gently apply 90–100 μl of blocking solution (PBS + 0.05% Triton ×100, 5% goat serum, 5% donkey serum) onto the coverslip and incubate for 1 h at 37°C. It is helpful to leave the coverslips on a Parafilm-covered surface during the blocking and subsequent incubations to avoid spillage of the solution from the coverslip; **2 Labelling with primary and secondary antibodies:** Incubate o/n at 4°C with the desired primary antibodies diluted in PBS containing 0.05% Triton X-100 and 2.5% donkey serum. Wash 3 times in PBS containing 0.1% Triton X-100 and twice in PBS alone. Subsequently, incubate the cells with the specific Alexa-conjugated secondary antibodies for 1 h at 37°C. Wash 5 times in PBS and rinse the covers in MilliQ water to remove excess salts.

### FM1-43 experiment and *post-hoc* immunocytochemical analysis

**Figure 3:**
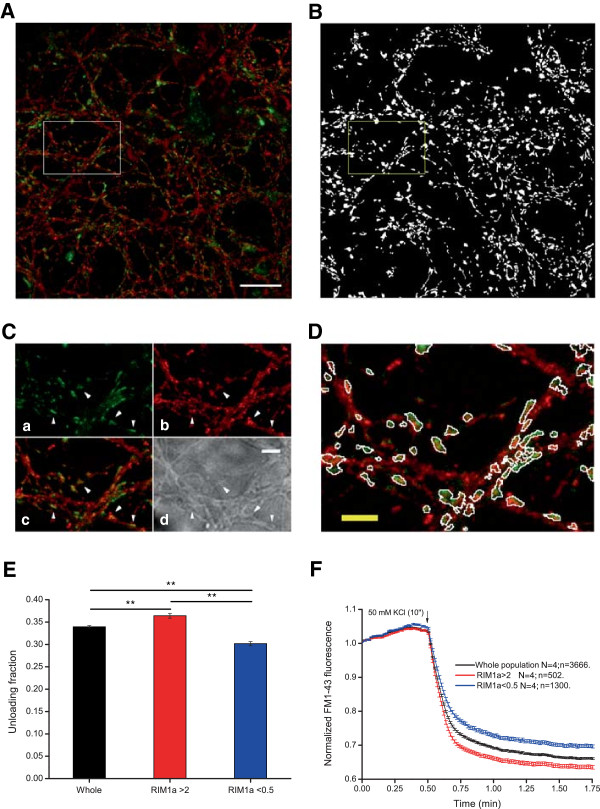
**RIM1α levels correlate with synaptic efficiency. A)***Post-hoc* immunocytochemical images of FM1-43 loaded boutons in green and anti-RIM1α in red. **B)** Binary mask of the field imaged in **A)** obtained using IgorPro software for processing the experiments. **C)** Higher magnification detail of the boxed area in **A)** showing FM1-43 staining (a), RIM1α labelling (b), the merged image (c) and phase contrast image (d). Arrowheads indicate different synaptic boutons. Scale bar = 5 μm. **D)** Area boxed in A after superimposition of the detail boxed in B. Note the absence of “dead” pixels in the different ROIs in both channels (FM1-43 and RIM1α). **E)** Mean FM1-43 unloading values of the whole population of synaptic boutons (black), the subpopulation of synaptic boutons whose RIM1α content is higher than 2 (red) and the subpopulation of those ROIs which RIM1α content is lower than 0.5 (blue). **F)** Normalized FM1-43 unloading according to RIM1α IR values: whole population in black, subpopulation of synaptic boutons with IR > 2 in red and subpopulation of synaptic boutons with IR value lower than 0.5 in blue. Unloading fractions in **E** and traces in **F** are means of 3666 synaptic boutons from 4 covers (whole population), 502 boutons from 4 covers (>2) and 1300 boutons from 4 covers (<0.5). Scale bar = 25 μm. One way ANOVA followed by Bonferroni’s test for means comparison was performed. Significance was considered when p < 0.05.

To carry out a semi-automated and unbiased assay of synaptic efficiency on populations of thousands of individual nerve terminals and to specifically correlate each individual response with a given IR value, we employed a routine previously described by Bergsman et al. 2006 [46]. This processing technique is extremely useful to avoid manual data selection and analysis, thereby facilitating the statistical analysis of large populations of individual nerve terminals. This routine can be applied using IgorPro software and the data was analyzed as described previously [1]. Briefly, to ensure a minimum quality of the defined/selected regions of interest (ROIs), this routine uses three parameters to classify the responses: the slope of the baseline, the extent of dye unloading, and the coefficient of variation of the baseline. This routine also renders a mask that can be used for further analysis and it provides a set of images in which background subtraction has been performed automatically. The protocol used is described below in detail, and corresponds to step 3 in Figure 1; **1 Data Processing:** Collect the data and save the multidimensional “*”.TIFF file as an image sequence in ImageJ (File > Save as > Image Sequence). Process the image sequence as indicated in Bergsman et al. 2006 [46]. The specific parameters used in this analysis are described in Additional file 4: Figure S3A; **2 Data Transfer:** Once the analysis has finished check “leave folder alone” when prompted, which will save the processed (background subtracted) images in the same folder in which the experimental data is stored. Next, select Data > Browse Waves and save the QualitySegment.ibw file. This file is an Igor Binary Wave (“.”.ibw) file that can be imported as an 8-bit image into ImageJ software (File > Import > Raw), and it is a binary drawing of the mask used for ROI analysis during the experiment. The settings for importing should be adjusted according to the size of the images acquired during the experiment (1004 × 1002 in this protocol: Additional file 4: Figure S3B); **3 Serial Reconstruction:** Build a serial reconstruction of the lower magnification (20×) images acquired at the end of the FM1-43 experiment (step 5 in section FM1-43 live cell imaging and Additional file 1: Figure S1). This operation can be performed automatically with Adobe Photoshop CS3 by loading the cell phase contrast images (File > Automate > Photomerge), although other software packages can also be used to this end; **4 Image localization:** Visually search for the exact field examined in the functional experiment by manually surveillance with minimal clear light intensity to avoid photobleaching of the labelled secondary antibodies. This may take several minutes, depending on the expertise of the investigator; **5 Image acquisition:** Once the field has been found, acquire immunofluorescence images of the field monitored during the experiment at the same magnification (60× in the present protocol). Characteristic hallmarks of the field are useful to help manually align the coverslip before acquiring the immunofluorescence images, taking the FM1-43 experiment as a reference. This operation minimizes the subsequent digital alignment and reduces the number of lost (non-matching) ROIs during digital processing (step 4 in Figure 1); **6 Background Subtraction:** Perform a rolling ball background subtraction on the images acquired using a 12-pixel ball radius [31]. Take the FM1-43 image as a reference and after merging the channels (Image > Color > Merge Channels), align the images using the Image J Align RGB planes plug-in (http://www.dentistry.bham.ac.uk/landinig/software/software.html). Once aligned, split the channels (Image > Color > Split channels) and save the aligned images for posterior analysis (steps 5 and 5′ in Figure 1); **7 Establish ROIset:** Generate a ROIset in ImageJ from the “.”.ibw file (Additional file 4: Figure S3B). Import the mask and render a binary drawing (Process > Binary > Make binary). After alignment, some of the edges are usually missing from the immunofluorescence images and the corresponding ROIs must be removed from the binary image. This can be done easily by selecting the missing area in the immunofluorescence image and restoring that selection to the binary drawing. Finally, render the ROIset (Analyze > Analyze particles; tick the options appearing in Additional file 4: Figure S3C) and save this ROIset as a “.”.zip file; **8 Data Acquisition:** Obtain the data from the different images, for which we recommend using the automatically generated background-subtracted images obtained from the IgorPro analysis in order to minimize user manipulation of the images. This is achieved by automatically importing the images generated from the folder in which the experiment was saved (follow the steps indicated in Additional file 4: Figure S3D). After superimposing the ROIset over the images to be analysed, go to the ROIset menu and click More > Multi measure. In the present analysis, integrated densities were used to provide a cumulative measure of the immunoreactivity for each individual ROI [1]. List the results obtained and copy them into a different worksheet (OriginPro 8.0 was used in the present study). Ensure that the same naming system is used for the columns in each worksheet so that the immunoreactivity values will match the kinetic values of each of the ROIs. Subsequently, you can check that the three images are properly aligned by plotting IR in channel 1 (Alexa 488, corresponding to RIM1α immunolabelling in this example) versus IR in channel 2 (Alexa 594, corresponding to CB1R immunolabelling in this case: Figure 2B and 2D). Generate a merged image in which the alignment can also be checked by eye; a line plot of the three channels is also helpful to assess the alignment (Additional file 5: Figure S4A and B); **9 Normalization:** Normalize the IR values by dividing by the mean IR value of each of the channels. Although immunoreactivity levels are not linearly related to the amount of protein [27], by normalizing it is possible to perform a comparative analysis between the edges of the distribution in a given population of synapses (Figure 2E). Moreover, this data processing allows the kinetic responses obtained during the FM1-43 experiment to be sorted blindly into different groups according to the intensity of IR. Microsoft Excel can be used to generate logic value codes (0 and 1) to blindly sort the functional responses (step 6 in Figure 1). In the present study we sorted the responses into three groups: the whole population of responses; the responses of ROIs with IR intensity more than twice the mean value; and the responses of ROIs in which IR intensity was less than half the mean value. The mean values of the three groups can then be plotted. An example of an individual experiment with a positive kinetic segregation is shown in Figure 2F (see also Figure 3E and 3F). For multiple comparisons, we used ANOVA followed by a Bonferroni test to compare the means.

## Results and discussion

### The intensity of RIM1α, unlike that of Munc13-1, is a *bona fide* indicator of synaptic efficiency

Although for decades primary neuronal cultures have been used to study various aspects of synaptic physiology, including pre- and postsynaptic function [6,13,22,32,33], synaptopathies and intersynaptic trafficking [34-37], the relationship between certain parameters of synaptic activity and protein content remains unclear. While it is widely accepted that different proteins carry out specific activities (*e.g.*, exo- and endocytic proteins mediate exo- and endocytic processes, respectively), few studies have demonstrated a quantitative correlation between synaptic function and protein content. Several aspects of presynaptic function have recently been correlated with protein levels at a given release site [7], and interference with the dynamics of protein synthesis/degradation has been shown to modulate synaptic strength [4,6,38]. For example, RIM1α levels are linearly related to the release probability (Pr) of local axon collaterals of CA3 [7], and presynaptic efficiency can be bidirectionally modulated by modifying RIM1α levels [38]. To determine the extent to which differences in the magnitude of responses between individual nerve terminals are caused by variations in protein content, we developed a method in which *post-hoc* immunolabelling is combined with (but not limited to) optical tracking of the synaptic vesicle (SV) cycle with FM1-43, this is a powerful tool to study the correlation between protein content and synaptic function, in this case vesicular release, a relationship that has remained undefined for decades in studies of synaptic function. Using the extensive body of published data relating to the exocytotic steps of the SV cycle (for review see [39]), we investigated whether the levels of two key proteins involved in the formation of exocytic complexes (Munc13-1 and RIM1α) are correlated with presynaptic activity, as measured by vesicular release.

Our results demonstrate that synaptic levels of RIM1α are positively correlated with FM1-43 unloading, which is a direct measure of vesicular reuse and release (Figure 3E and 3F). In this context we have found that those synaptic boutons whose RIM levels are higher than 2 times the mean IR value yielded by the whole population of boutons, are more efficient in terms of FM1-43 release. The opposite effect was found in the subpopulation of boutons with RIM1α levels lower than 0.5 times the mean IR value (Unloaded fraction; Whole population: 33.95 ± 0.27%, N = 4, n = 3666; RIM1α IR > 2: 36.41 ± 0.53%, N = 4, n = 502, **p < 0,01; RIM1α IR < 0.5: 30.18 ± 0, 47%, N = 4, n = 1300, **p > 0,01; ANOVA followed by Bonferroni’s Test for means comparison). These results are consistent with previous findings [4,7,13,29] and they validate the use of this technique. Based on these findings, it is possible to classify the functional responses of the FM1-43 experiment blindly into categories of increasing efficiency by sorting the ROIs according to the intensity of RIM1α IR. RIM1α is a pivotal protein in the arrangement of presynaptic active zones [26] and it participates in a complex interaction network along with other presynaptic proteins, such as the vesicular protein Rab3 [40] and the priming factor Munc13-1 [8]. Another important role of RIM1α is to tether calcium channels to presynaptic active zones via its PDZ domain [13,29,41], a function that may be critical in coupling exocytosis to calcium influx. RIM1α also undergoes PKA-dependent phosphorylation [42] and ubiquitin-dependent degradation via the E3 ubiquitin-ligase SCRAPPER [38]. Low levels of RIM are associated with low mEPSC frequencies and low calcium sensitivity [38], while high levels are linked with an increased Pr [7]. These data support the hypothesis that RIM levels can dictate the release properties of different synapses, serving as a source of variability among populations of synapses.

**Figure 4:**
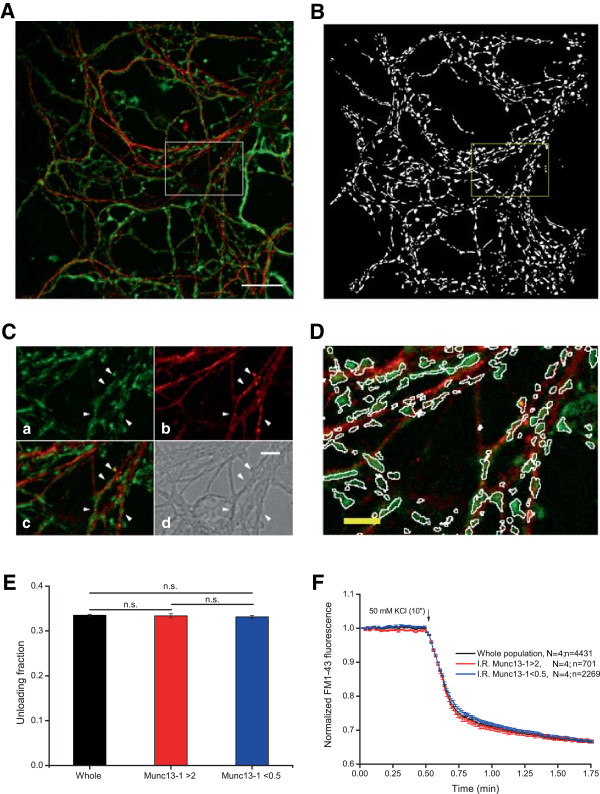
**Munc13-1 levels do not correlate with synaptic efficiency. A)***Post-hoc* immunocytochemical images of FM1-43 loaded boutons in green and anti-Munc13-1 in red. **B)** binary mask of the field imaged in **A)** obtained using IgorPro software for processing the experiments. **C)** Higher magnification detail of the boxed area in **A)** showing FM1-43 staining (a), Munc13-1 labelling (b), the merged image (c) and phase contrast image (d). Arrowheads indicate different synaptic boutons. Scale bar = 5 μm. **D)** Area boxed in A after superimposition of the detail boxed in B. Note the absence of “dead” pixels in the different ROIs in both channels (FM1-43 and Munc13-1). **E)** Unloading fraction values of the whole population of synaptic boutons (black), the subpopulation of synaptic boutons whose Munc13-1 content is higher than 2 (red) and the subpopulation of those ROIs which Munc13-1 content is lower than 0.5 (blue). **F)** Normalized FM1-43 unloading according to Munc13-1 IR values: whole population in black, subpopulation of synaptic boutons with IR > 2 in red and subpopulation of synaptic boutons with IR value lower than 0.5 in blue. Unloading fractions in **E** and traces in **F** are means of 4461 synaptic boutons from 4 covers (whole population), 761 boutons from 4 covers (>2) and 2261 boutons from 4 covers (<0.5). Scale bar = 25 μm. One way ANOVA followed by Bonferroni’s test for means comparison was performed. Significance was considered when p < 0.05.

Another important protein involved in the formation of exocytic complexes is the mammalian homologue of UNC13, the multidomain Munc13-1 protein, although we were unable to detect a positive correlation between Munc13-1 levels and synaptic efficiency (Figure 4E and 4F, Unloaded fraction; Whole population: 33.51 ± 0.20%, N = 4, n = 4431; Munc13-1 IR > 2: 33.37 ± 0,47%, N = 4, n = 701, non significant; Munc13-1 IR < 0.5: 33.15 ± 0.31%, N = 4, n = 2269, non significant). While unexpected, this result adds further weight to the positive correlation between RIM1α levels and FM1-43 unloading, indicating that the differences observed were not due to image processing or analysis. However, mechanistic differences between these two proteins could account for the failure to identify a correlation between Munc13-1 levels and vesicular release. Munc13-1 exists as a homodimer that must heterodimerize with RIM1α to carry out its priming function [14]. Moreover, it can also be regulated by diacylglycerol [16], calmodulin [43] and calcium [44]. Diacylglycerol binding to Munc13-1 translocates the protein to the membrane, where it can carry out its priming function [45]. Hence, activation of the priming function of Munc13-1 involves several regulatory steps. We propose that the total levels of Munc13-1 (as determined by IR measurements) do not allow us to distinguish between active and inactive pools of this protein, which might explain the lack of a correlation between Munc13-1 IR and vesicular release. Moreover, we cannot rule out the possibility that poor antibody sensitivity or specificity accounts for the absence of a correlation between global Munc13-1 levels and synaptic efficiency. However, both antibodies identified a single band of the expected molecular weight when assessed in immunoblots (Additional file 2: Figure S2).

In the present study, we present a method that can be used to correlate a functional parameter of synaptic physiology (SV release) with the levels of different proteins present at a given release site. The main advantage of this technique is the reduced user intervention during data processing, as manual selection and drawing of the different ROIs is avoided. In most studies assessing synaptic function using image techniques, ROIs are user-defined and as such, they represent a source of potential bias. This bias is not only due to the poor sensitivity of the human eye compared with processing software but also, because the ROI shape does not exactly match that of the synaptic boutons, resulting in the inclusion of dead pixels in the ROIs defined, which can in turn affect the numerical data obtained. Moreover, manual selection usually renders fewer ROIs, which is not optimal for high-level statistical tests. In our protocol we employed an automated routine [46] to generate a mask that includes several hundred ROIs per experiment, the shape of which exactly matches that of the FM1-43 puncta and the IR puncta in the *post-hoc* images. This routine also generates a set of images in which the background is subtracted automatically and that can be used for analysis. The validity of this method is corroborated by the correlation observed between RIM1α levels and the effectiveness of vesicular release, consistent with previous data [4,7,38]. Moreover, the responses are blindly sorted according to their IR intensity, thereby eliminating another source of bias. This protocol is not limited to the assessment of synaptic activity using FM1-43, and it can be used to correlate semiquantitative IR data with the calcium influx, measures of the exo/endocytic cycle with pHluorins, or electrophysiological recordings. This technique could also be further improved by incorporating super-resolution microscopy techniques such as STED [47,48] or STORM [49].

## Conclusions

Altogether, our results indicate that nerve terminal content of RIM1α strongly correlates with the release capacity of the nerve terminal measured with FM1-43, while no such a correlation was found with Munc13-1. This finding point out to RIM1α as a crucial factor in determining synaptic efficiency and demonstrate the usefulness of this technique to analyse the molecular differences underlying the heterogeneous responses exhibited by neuronal networks.

## Competing interests

The authors declare that they have no competing interests.

## Authors’ contributions

JRF, JSP and MT conceived the study. JRF, BA and DBM designed experiments, and were responsible for collect, analyse and interpret the data; JRF and JSP wrote the manuscript. MT and JSP acquired funding necessary for the completion of the study. All authors read and approved the final manuscript.

## Supplementary Material

Additional file 1: Figure S1 Serial reconstruction of the imaged field. **A)** Synaptic boutons loaded with FM1-43 dye before (t_o_) and after (t_end_) stimulation with potassium chloride; contrast phase images of the same experiment field (white box) at different magnifications: 60× **(B)**, 40 × **(C)** and 20× **(D)**. **E)** Serial reconstruction of 20× cell phase images of the field monitorized during the experiment (white box) and surrounding areas. **F)** Contrast phase image al 20× magnification showing the field where the experiment was performed after fixing and labelling with the different antibody (matching field). Note the presence of distinctive hallmarks. Click here for file

Additional file 2: Figure S2 Western blot of RIM1α and Munc13-1 confirmed specificity of both antibodies. **A)** Western Blot of Munc13-1 (top green), β-Tubulin (bottom, red) and synaptophysin (bottom, green), showing a single band for Munc13-1 with the expected molecular weight. **B)** Western blot of RIM1α (green, top) and β-Tubulin (green, bottom) showing that both antibodies recognize a single band with the expected molecular weight. Click here for file

Additional file 3 Supplementary methods. Click here for file

Additional file 4: Figure S3 FM1-43 experiment analysis routine using IgorPro software and ImageJ. **A)** Parameters used in IgorPro interface to automatically analyze the FM1-43 experiment. This software renders the mask and a set of background subtracted images. **B)** Import of the QualitySegment.ibw file in ImageJ software to draw a binary mask. The size of the image has to fit with the original images acquired during the experiment. **C)** Generation of the ROIset with ImageJ software by analyzing particles present in the mask. **D)** Import of the set of background-subtracted images for further analysis of the experiment with OriginPro. Click here for file

Additional file 5: Figure S4 Alignment of FM1-43 with IR puncta after *post-hoc* ICC. **A)** Immunocytochemical images of FM1-43 in blue (pseudo colour), Alexa 488 (green) and Alexa 594 (red). Upper panels show ROIset superimposition and lower panels show line plot along a fiber. Note that the different ROIs are well fitted to the puncta in the different channels, this step is useful to visually check the alignment of the three channels. **B)** Arbitrary fluorescence units plot over the three channels of the ROIs indicated in **A)**. Click here for file
