# Supplementary figures and images for: Studying synaptic efficiency by post-hoc immunolabelling
Source: BMC Neurosci. 2013 Oct 18;14:127. doi: 10.1186/1471-2202-14-127 (PMC3854067; doi:10.1186/1471-2202-14-127)

Ramírez-Franco et al., Supplementary Figure 1

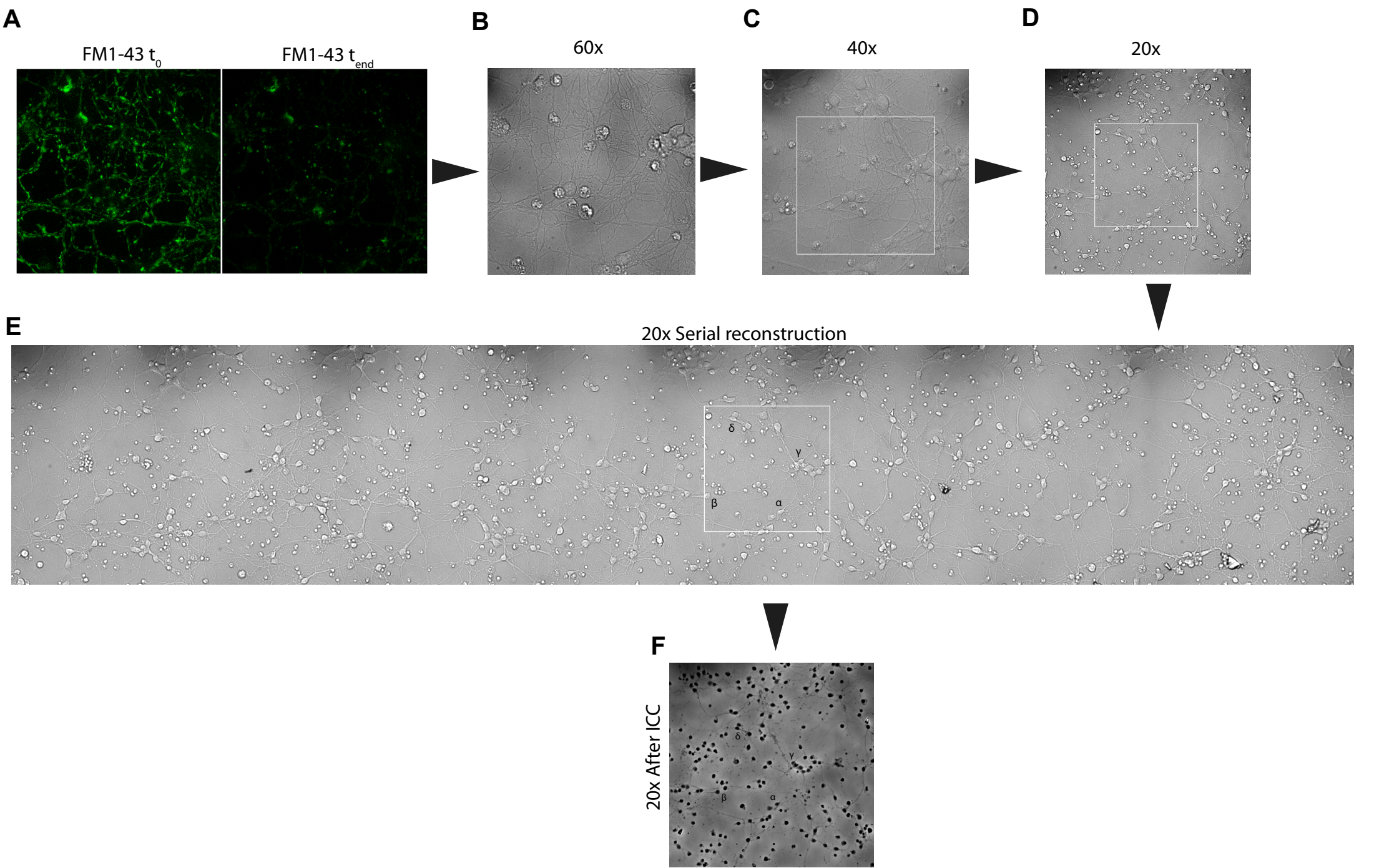

Supplement: Additional file 1: Figure S1 — Serial reconstruction of the imaged field. A) Synaptic boutons loaded with FM1-43 dye before (to) and after (tend) stimulation with potassium chloride; contrast phase images of the same experiment field (white box) at different magnifications: 60× (B), 40 × (C) and 20× (D). E) Serial reconstruction of 20× cell phase images of the field monitorized during the experiment (white box) and surrounding areas. F) Contrast phase image al 20× magnification showing the field where the experiment was performed after fixing and labelling with the different antibody (matching field). Note the presence of distinctive hallmarks. [file 1471-2202-14-127-S1.pdf]

A

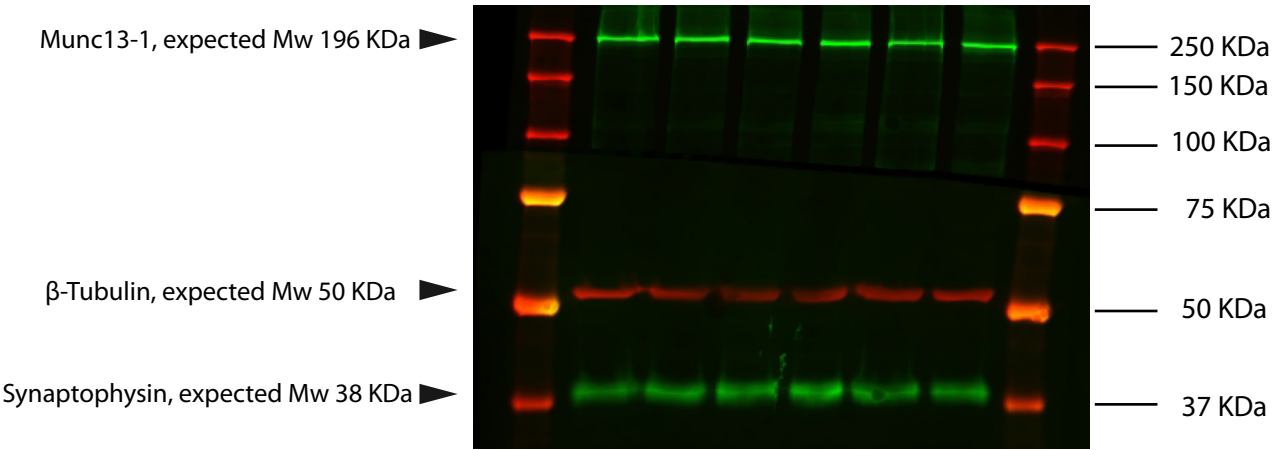

B

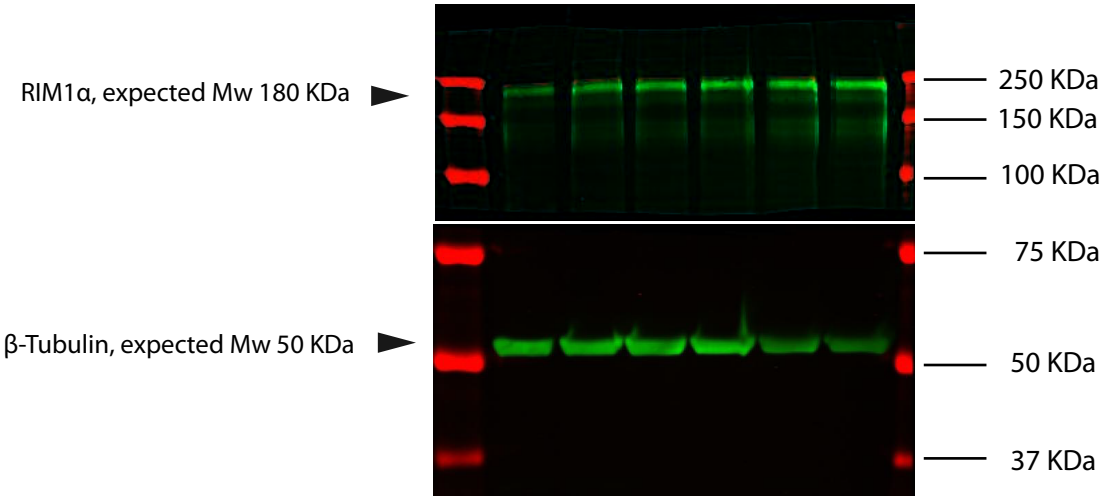

Supplement: Additional file 2: Figure S2 — Western blot of RIM1α and Munc13-1 confirmed specificity of both antibodies. A) Western Blot of Munc13-1 (top green), β-Tubulin (bottom, red) and synaptophysin (bottom, green), showing a single band for Munc13-1 with the expected molecular weight. B) Western blot of RIM1α (green, top) and β-Tubulin (green, bottom) showing that both antibodies recognize a single band with the expected molecular weight. [file 1471-2202-14-127-S2.pdf]

Ramírez-Franco et al., Supplementary Figure 4

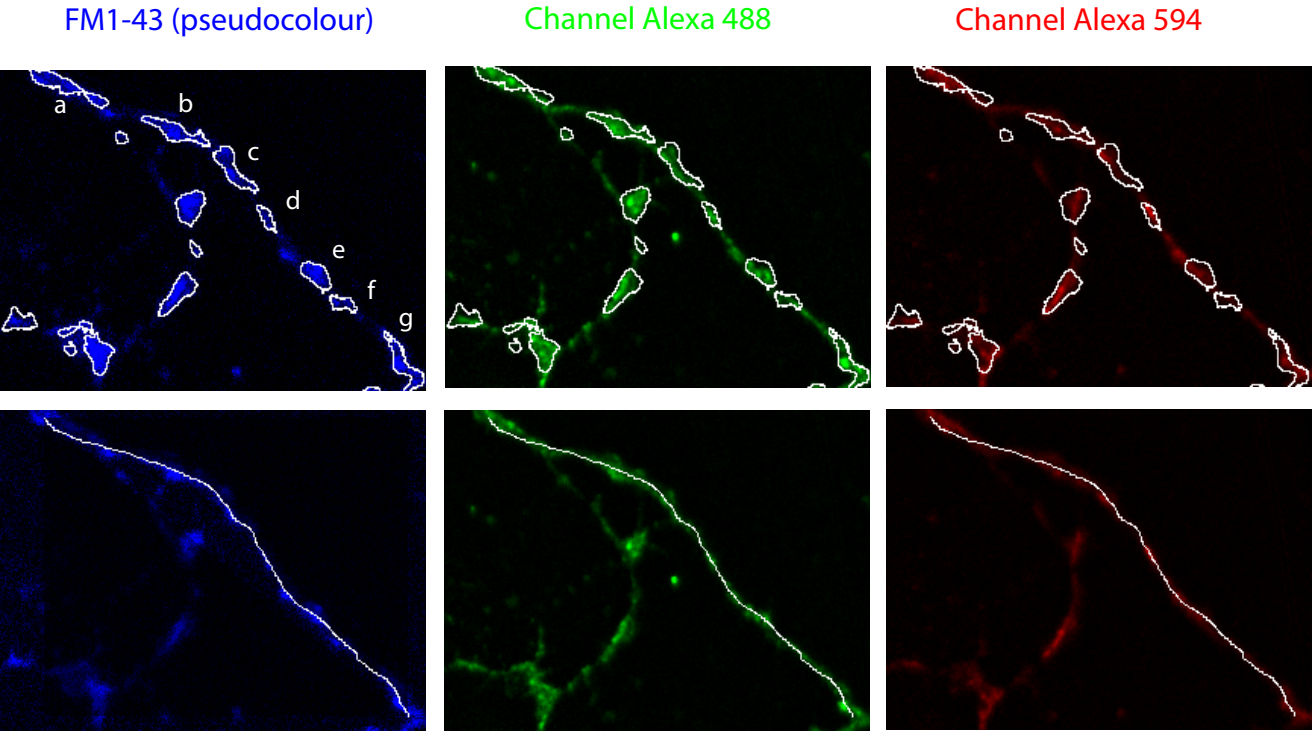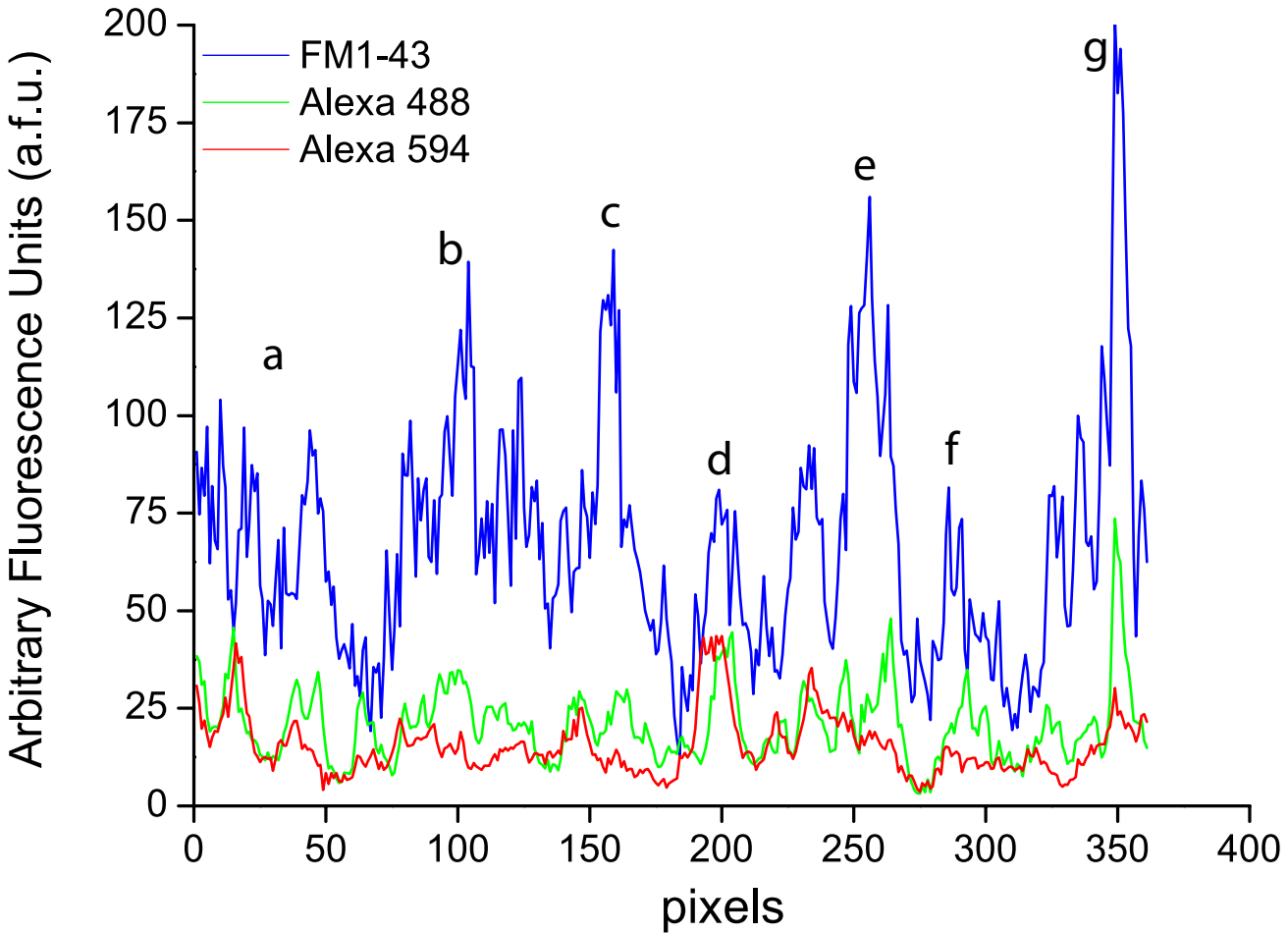

Supplement: Additional file 5: Figure S4 — Alignment of FM1-43 with IR puncta after post-hoc ICC. A) Immunocytochemical images of FM1-43 in blue (pseudo colour), Alexa 488 (green) and Alexa 594 (red). Upper panels show ROIset superimposition and lower panels show line plot along a fiber. Note that the different ROIs are well fitted to the puncta in the different channels, this step is useful to visually check the alignment of the three channels. B) Arbitrary fluorescence units plot over the three channels of the ROIs indicated in A). [file 1471-2202-14-127-S5.pdf]
