# Supplementary material for: Studying synaptic efficiency by post-hoc immunolabelling
Source: BMC Neurosci. 2013 Oct 18;14:127. doi: 10.1186/1471-2202-14-127 (PMC3854067; doi:10.1186/1471-2202-14-127)
Supplement: Additional file 3 — Supplementary methods. [file 1471-2202-14-127-S3.doc]

**Supplementary methods**

**Western Blotting.** The protein extracts from cultured cerebellar granule cells at 7 Days *In Vitro* were separated on 8-10 % sodium dodecyl sulphate-polyacrylamide gels and electrophoretically transferred to nitrocellulose membranes (Hybond, Amersham biosciences) as described previously [1]. The membranes were probed with the appropriate primary antibodies, rabbit polyclonal anti-RIM1α (1:500, ref. 140 003, Synaptic System), mouse monoclonal anti-Munc13-1 (1:200, ref. 126 111, Synaptic Systems), rabbit polyclonal anti-synaptophysin 1 (1:1000, ref. 101 002, Synaptic System) and mouse monoclonal-β-tubulin (1:2000, ref. T0198, Sigma). After several washing they were incubated with the corresponding IRDye-labeled secondary antibody; goat polyclonal anti mouse IRDye 680, (1:200, ref. 926-32220, Li-Cor Biosciences), goat polyclonal anti mouse IRDye 800CW (1: 200, ref. 926-32210, Li-Cor Biosciences) and goat polyclonal anti rabbit IRDye 800CW (1:200, ref. 926-32211, Li-Cor Biosciences), were used . Blots were scanned in an Odyssey Infrared imaging system. β-Tubulin or Synaptophysin were used as loading controls.
