# Supplementary material for: Studying synaptic efficiency by post-hoc immunolabelling
Source: BMC Neurosci. 2013 Oct 18;14:127. doi: 10.1186/1471-2202-14-127 (PMC3854067; doi:10.1186/1471-2202-14-127)

Ramírez-Franco et al., Supplementary Figure 3

A

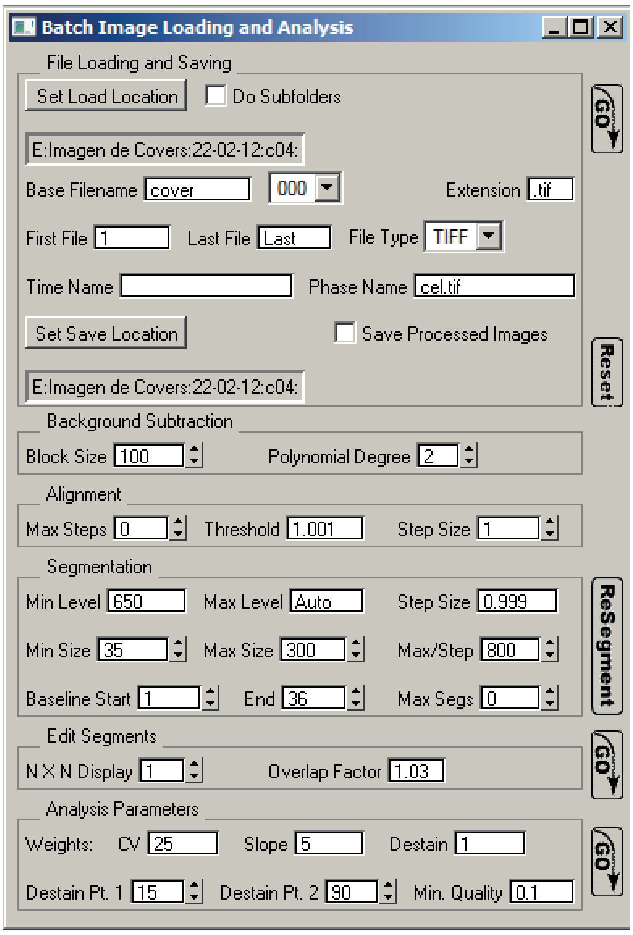

B

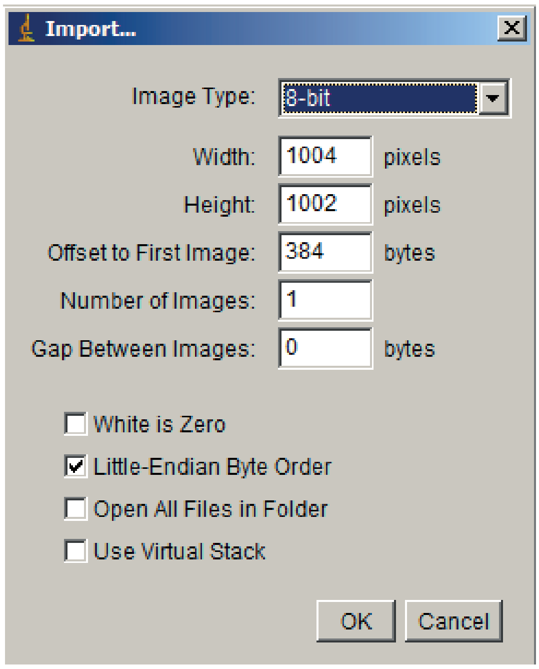

C

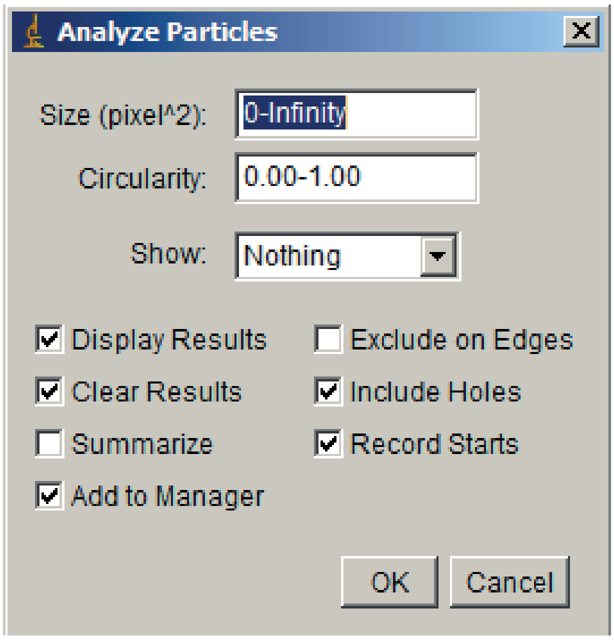

D

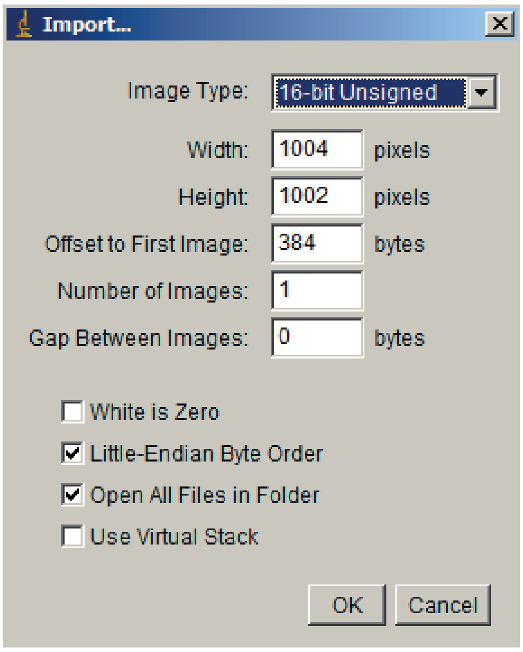

Supplement: Additional file 4: Figure S3 — FM1-43 experiment analysis routine using IgorPro software and ImageJ. A) Parameters used in IgorPro interface to automatically analyze the FM1-43 experiment. This software renders the mask and a set of background subtracted images. B) Import of the QualitySegment.ibw file in ImageJ software to draw a binary mask. The size of the image has to fit with the original images acquired during the experiment. C) Generation of the ROIset with ImageJ software by analyzing particles present in the mask. D) Import of the set of background-subtracted images for further analysis of the experiment with OriginPro. [file 1471-2202-14-127-S4.pdf]
